# Hypercompetition, score compression and AI co-authorship: a perfect storm for grant success rates

**DOI:** 10.1038/s44319-026-00779-4

**Published:** 2026-04-15

**Authors:** Shina Caroline Lynn Kamerlin

**Affiliations:** Georgia Institute of Technology, School of Chemistry and Biochemistry, Atlanta, GA USA

**Keywords:** Science Policy & Publishing

## Abstract

The use of AI in grant writing to address non-scientific selection criteria along with growing application numbers and score inflation have turned many research funding schemes efficiently into lotteries.

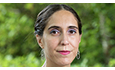

Hyper-competition, score inflation and the ascendancy of AI use in both grant writing and—although typically not permitted—peer review are putting severe pressure on grant success rates. This is particularly alarming as many funding agencies and programs have for years had extremely low success rates due to the increasing number of applications. For instance, the most recently announced success rates for European Research Council (ERC) Starting (2025), Consolidator (2025), Advanced (2024) and Synergy (2025) grants were 12.2, 11.2, 11, and 9.3%, respectively, and for the European Innovation Council (EiC) Pathfinder Grants, success rates currently are at ~2% (2025 call results). In this column, I will provide a concrete case study focusing on the Marie Skłodowska-Curie Actions (MSCA) postdoctoral Fellowships, illustrating how these factors can work together to create a perfect storm for prestigious funding schemes. It turns them effectively into grant lotteries at great cost for both individual researchers and for the system more broadly. While I use MSCA fellowships as a case study, the purpose is not to suggest that this scheme is particularly bad, but rather to showcase a problem that unfortunately affects scientific funding in general.

The results of the 2025 call for proposals for MSCA Postdoc Fellowships were released in February 2026, with €404.3 million awarded to 1610 postdoctoral researchers, out of a total of 17,066 applicants, of which 16,836 were eligible; this yields a success rate of 9.6%. In March 2026, the European Commission released the statistics for the 2025 MSCA doctoral programs call, awarding 141 doctoral programs out of 1616 applications received: a 8.7% success rate.

While these numbers seem similar to the success rates of ERC individual PI grants or national funding programs like the Swedish Research Council grants (~14% overall success rate, 2025 statistics), they hide the extreme scores needed to succeed in the MSCA program. To my shock, this year, a score of 96.4, which only recently would have been not only fundable but extremely competitive, was not high enough for a postdoc in my group to even make the reserve list. Indeed, looking at the score distribution provided in the 2025 MSCA postdoc fellowship call results, the cutoff scores for funding ranged from 93.4 (GF-ECO, small panel with only 15 proposals) to 97.6 (GF-CHE, another small panel with just 67 proposals). Typical panel cutoff scores were in the 96–97 range, and 6 out of 16 panels received 1699–3208 proposals (their cutoff scores ranged from 96.4 to 97). More alarmingly, these high cutoff scores were a result of substantive score compression: in multiple panels, more than 50% of proposals had a score of at least 89 —in an extreme case, GF-PHY, it was 76 proposals or 71.03% of proposals—and in the majority of panels (13/16), at least a 5th of proposals scored 93 or above.

This is problematic for multiple reasons: first, it indicates not just brutal funding cutoffs, but also severe score inflation, which makes it essentially impossible to distinguish between proposals of similar quality. As a result of the deluge of high scores, reviewers are not truly selecting the “best” proposals from the pile of applications. Indeed, reviewers are not great at distinguishing between high quality proposals more generally—this is often called the grey-zone problem, which is exacerbated by low funding rates. Some scholars have commented that NIH peer review percentile scores are poor predictors of productivity along with other related analyses. As a result, there is an argument to be made in favor of removing numerical scoring completely in a situation of severe score inflation.

This situation is further exacerbated by the fact that funding agencies are facing unprecedented and growing numbers of grant applications, putting tremendous pressure on their peer-review systems. Moreover, the increasing use of AI by both applicants and reviewers both increases the volume of grants being submitted and reduces the quality and integrity of peer review.

The latter point is a particular issue for programs such as the MSCA Postdoc Fellowships, where only 50% of the score focuses on excellence as a proxy for the quality of the research being proposed. And even this section contains non-research-based criteria such as quality of supervision, training, and two-way knowledge transfer between the researcher and the host: see the guidelines for applicants, available on the Funding and Tenders portal. In light of low success rates and reviewer’s difficulties to select the best proposals from a large pool of equally promising applications, there is an argument to be made in favour of complementing excellence with other, measurable attributes related to good scientific practice. However, while criteria, such a supervision, training or knowledge transfer, are certainly appropriate to assess a primary investigator or group leader who manages a research team, they are not necessarily suitable to evaluate the application by a PhD student or postdoc.

As mentioned earlier, this is further exacerbated by the increasing use of Large Language Models to write applications—and these AI chatbots are increasingly good at producing very convincing and elegant texts. My colleague Will Ratcliffe and I have previously written for this publication on the risks of using AI to prepare for academic interviews, because it takes away the personal aspect of the interview process and in doing so actually harms an applicant’s chances. In the context of an MSCA Fellowship and other related EU schemes, the non-research-based sections require very specific information to score highly, which can be achieved relatively effectively by an AI chatbot. For instance, I provided Google Gemini 3 with the prompt: “Write a two-way knowledge transfer section for an MSCA postdoc fellowship application by a hypothetical postdoc applicant coming from a European institution to my lab at Georgia Tech to work on evolutionary-based protein design. The applicant has a background in AI/ML for predicting protein conformational ensembles, and a computer science degree.” Gemini provided me with the following draft text:

*“Transfer of Knowledge to the Researcher (Host to Fellow)*

*The Fellow will be embedded within the Kamerlin Lab at the Georgia Institute of Technology, a world-leading environment for computational biophysics and enzyme evolution. Despite a strong background in Computer Science and AI-driven conformational ensembles, the Fellow requires “wet-lab informed” computational training to transition into a leader in evolutionary-based protein design*.

*Mechanistic Enzymology* & *Biophysics: The Fellow will receive advanced training in the physical principles governing enzyme catalysis and the role of conformational dynamics in protein evolution. This moves the Fellow beyond “black-box” ML predictions toward physically grounded design. […]*

*Transfer of Knowledge to the Host (Fellow to Host)*

*The Fellow brings a high-level technical skillset in Computer Science and Deep Learning that will significantly augment the Kamerlin Lab’s existing computational workflows*.

*AI-Driven Conformational Sampling: The Fellow will introduce state-of-the-art ML architectures (e.g., diffusion models or flow-matching) specifically designed to predict conformational ensembles rather than static structures. This will enhance the lab’s ability to model the “dynamic landscape” of enzymes, a known bottleneck in current protein design.”*

The LLM generated further text on computational efficiency & scaling, cross-pollination of methodologies, an implementation strategy, direct mentorship, collaborative benchmarking, and Open Science leadership that I have redacted for length. However, it is obvious that AI text bots are now able to create high-quality draft texts for both research and non-research-based aspects of a proposal. This means that an increasingly smaller percentage of each proposal is truly individual and contributes to the score inflation and brutal cutoffs observed in the 2025 call, where even fractions of points made the difference between funding and not funding a proposal.

There has been an increasing call for grant funding to be allocated by lottery; whether that is a good idea or not is beyond the scope of this column. I do want to point out though that, as outlined above, the current model is already an implicit lottery, and this situation is deteriorating with increasing numbers of applications and AI usage. The call for the 2026 MSCA Postdoctoral Fellowships  opened in April 2026. If current trends persist, it would not be unreasonable to expect more than 15,000 applications, many of which will be written with the help of LLMs. There is very little buffer for further score pressure, with funding cutoff scores at 95 or higher. For this and similar funding schemes to remain sustainable, it is clearly necessary to urgently rethink and change the scoring criteria and peer-review procedures.

## Supplementary information

Peer Review File

