# Supplementary material for: Hypercompetition, score compression and AI co-authorship: a perfect storm for grant success rates
Source: EMBO Rep. 2026 Apr 15;27(9):2145–6. doi: 10.1038/s44319-026-00779-4 (PMC13172394; doi:10.1038/s44319-026-00779-4)
Supplement: Supplementary file 1 — Peer Review File [file 44319_2026_779_MOESM1_ESM.pdf]

EMBO reports

Hypercompetition, Score Compression and AI Co-Authorship: A Perfect Storm for Grant Success Rates

Shina Caroline Lynn Kamerlin

Corresponding author(s): Shina Caroline Lynn Kamerlin (skamerlin3@gatech.edu)

Review Timeline:

Submission Date:	7th Apr 26
Accepted:	8th Apr 26

Editor: Holger Breithaupt

Transaction Report: This article type is not peer reviewed.
